# Standardized Rehabilitation or Individual Approach?—A Retrospective Analysis of Early Rehabilitation Protocols after Isolated Posterior Cruciate Ligament Reconstruction

**DOI:** 10.3390/jpm12081299

**Published:** 2022-08-08

**Authors:** Clemens Memmel, Matthias Koch, Dominik Szymski, Lorenz Huber, Christian Pfeifer, Christian Knorr, Volker Alt, Werner Krutsch

**Affiliations:** 1 Department of Pediatric Surgery and Orthopedics, Clinic St. Hedwig, Barmherzige Brueder Regensburg, KUNO Pediatric University Medical Center and FIFA Medical Center of Excellence, University Medical Center Regensburg, 93053 Regensburg, Germany; 2 Department of Trauma Surgery and FIFA Medical Center of Excellence, University Medical Center Regensburg, 93053 Regensburg, Germany; 3 Department of Orthopedic Surgery and Traumatology, Kantonsspital Baselland, Bruderholz, 4101 Basel, Switzerland; 4 Department of Trauma Surgery, InnKlinikum Altoetting, 84503 Altoetting, Germany; 5 SportDocs Franken, 90455 Nuremberg, Germany

**Keywords:** posterior cruciate ligament reconstruction, rehabilitation, weight bearing, range of motion, standardized rehabilitation

## Abstract

(1) Background: Isolated posterior cruciate ligament (PCL) tears represent a severe type of injury. In hospitals, PCL reconstruction (PCL-R) is less frequently performed than other types of knee surgery. It is unclear whether there is consensus among surgeons on how to perform rehabilitation after PCL-R or if there are different, more individual approaches in daily routines. (2) Methods: Rehabilitation protocols and their main criteria (the progression of weight bearing and range of motion, the use of knee braces, rehabilitation training, and sports-specific training) were retrospectively analyzed after PCL-R. (3) Results: Only 33 of 120 (27.5%) analyzed institutes use rehabilitation protocols after PCL-R. The applied protocols showed vast differences between the individual rehabilitation criteria, especially with regard to the progression of weight bearing and the range of motion. The only standardized recommendations were the obligatory use of knee braces and the general restriction of weight bearing and range of motion immediately post-surgery. Therefore, because of the lack of a consensus about a standardized rehabilitation protocol after PCL-R, no recommendation can be made on one particular protocol. (4) Conclusion: There is no acknowledged standardized rehabilitation protocol after PCL-R. In clinical practice, recommendations are influenced by, i.a., surgeons’ opinions and experience. The lack of scientific evidence on a particular standardized rehabilitation protocol after PCL-R suggests that rehabilitation protocols need to be tailored to the individual patient.

## 1. Introduction

The posterior cruciate ligament (PCL) extends from the lateral aspect of the medial femoral condyle, adherent to the articular cartilage, down to the PCL facet of the posterior tibia, approximately 1 to 1.5 cm below the tibial plateau. Its course is intrasynovial but extraarticular [1,2,3]. The main function of the PCL is to limit posterior tibial translation relative to the femur, with an increasing effect the higher the flexion in the knee joint [1,4]. Other tasks, albeit to a lesser extent, include restraining internal and external as well as varus–valgus rotation [1,5]. As previously described, the biomechanical stress on the posterior cruciate ligament increases with the amount of flexion in the knee joint, when the knee joint is passively moved. During active movement, certain muscle groups play a special role, which, when activated, load or unload the posterior cruciate ligament. While activation of the gastrocnemius muscles significantly increases the tension on the posterior cruciate ligament at flexion from 40°, the quadriceps muscles relieve the posterior cruciate ligament at angles between 20 and 60° of flexion. In turn, when the hamstring muscles are activated, this increases the tension on the posterior cruciate ligament just at angles of 70 to 110 degrees of flexion [6]. These anatomical and biomechanical principles must always be present not only if one wants to perform sufficient PCL-R but also postoperative rehabilitation.

Even in patients with asymptomatic, isolated PCL injuries, abnormal knee kinematics can be found during functional activities such as stair ascent and running [7]. In general, tears of the PCL are far less frequent than tears of the anterior cruciate ligament (ACL). The incidence of complete isolated PCL ruptures is 2 per 100,000 [8]. PCL ruptures occur less often isolated than in combination with multi-ligament injuries of the knee or with cartilage defects [9,10,11]. In 16% of isolated PCL reconstruction (PCL-R), meniscus injury could also be detected. The majority (64%) of isolated PCL ruptures that require PCL-R result from sports injuries. In comparison, in isolated ACL reconstruction, the mechanism of injury is 89% sports-related [11]. Even though the rupture of the PCL is often well tolerated clinically, such ruptures significantly increase the biomechanical stress on the medial and retropatellar articular cartilage and, thus, the risk of medial and retropatellar gonarthrosis [8,12,13,14]. Studies have shown that conservative treatment of acute isolated PCL ruptures may achieve good functionality and stability of the injured knee joint [15,16,17,18]. Since the development of modern surgical techniques, however, arthroscopically assisted surgery has become increasingly sufficient even in the case of acute isolated PCL ruptures with significant posterior tibial translation or chronic PCL tears with symptoms such as problems with deceleration or incline descending and posterior tibial translation [19,20]. In addition, long-term outcome measures of PCL-deficient knees and the role of the PCL in physiological knee biomechanics have become of increasing importance, so that PCL-R has become a suitable therapy for isolated PCL injury [4,21]. Still, the ideal management of isolated PCL injuries, that is conservative therapy vs. surgery and the choice of surgical technique, is still controversially discussed [22].

The rehabilitation phase must be at least as important in the overall treatment of the patient and must be accompanied in a similar way as the surgery itself. Its purpose is not only to protect the surgical result, but also, among other things, to restore the proprioceptive, coordinative, and conditional abilities lost since the injury. During the month-long rehabilitation phase, the patient should not only be accompanied by the orthopedic surgeon, but rather by a rehabilitation team, which, in addition to the patient and surgeon, also consists of a physiotherapist and at best people from the home environment (e.g., parents and partner). There should also be regular exchanges between the patient, the orthopedic surgeon, and the physiotherapist, not only to prevent possible undesirable developments in rehabilitation, but also to document progress and thus keep the patient motivated to continue. Because of a mostly relatively young, sportive patient group with high pre-injury activity levels, the aims of surgery and the following rehabilitation phase are set high. In principle, the pre-injury activity level of the injured person as well as the demand on the quality of life, including the participation in sports after the injury, should be considered when deciding on conservative or surgical therapy. The same principle applies to the rehabilitation phase. Adequate rehabilitation is the key factor for graft healing (particularly of hamstring grafts) and for improving patient outcome. Yet, standardized post-treatment protocols are often used to guide patients through the rehabilitation process and to instruct their physical therapists on weight bearing, the range of motion (ROM), and the begin of rehabilitative training. As recently shown, post-treatment protocols vary significantly, even for well-established and frequently used surgical therapies of the knee joint, such as ACL reconstruction [23] or cartilage-reparative procedures [24].

Based on this finding, the aim of this study is to answer the question whether there exists a standardized approach or if there are different and more individual approaches among orthopedic surgeons on how to perform postsurgical rehabilitation after isolated PCL-R in daily routines.

## 2. Materials and Methods

Design—In a retrospective cohort study, the early rehabilitation after PCL-R was analyzed in 120 institutes for orthopedic surgery. The medical device company OPED (GmbH, Valley, Germany) developed a tabular template for creating a post-treatment protocol after knee surgery (PCL R). This template was offered to orthopedic departments and orthopedic outpatient centers in German-speaking countries for free, so that associated orthopedic surgeons may create their own individual, but for their own institution standardized, post-treatment rehabilitation protocol using the criteria described in Table 1. OPED itself was not involved in the content of the rehabilitation protocols. All protocols were collected and blinded for study purposes. The investigated protocols contain general early rehabilitation recommendations made by orthopedic surgeons that were given to the patients and their physical therapists. The investigation focused on the availability of rehabilitation protocols after PCL-R and the criteria of early rehabilitation as described in Table 1.

Participants—For this qualitative study, we analyzed early rehabilitation protocols after knee surgery (PCL R) that are currently used in daily routines in German-speaking countries (Germany, Austria, and Switzerland). The protocols originated from 120 different orthopedic institutions, of which 63 were outpatient centers, 57 clinical centers, and 4 university medical centers.

Measures—This study focuses on the early rehabilitation phase. This phase can basically be divided into three episodes, each with its own goals. Within knee surgery, these phases are quite similar and are not necessarily PCL-specific. The timing of the phases, in turn, varies individually. The first episode includes the period immediately following surgery. It is also called the acute, inflammatory, or early protective phase. The therapeutic goals are to relieve pain, reduce swelling, and maintain or increase the mobility of the knee joint, especially the patellofemoral joint. The second episode is a transitional phase. It is characterized by a progressive increase in all dynamic modules of post-treatment. This mainly concerns the modules of loading and ROM. In addition, there is the restoration of coordinative abilities. Therefore, the start of rehab training usually falls into this phase. At the end of the second episode, full weight bearing, and the full ROM should be achieved. The third episode of early rehabilitation is the remodeling phase. Its therapeutic goal is to strengthen the important muscle groups and increase endurance with a view to mastering everyday skills. Everyday motor skills should be restored at the end of this episode. During this phase, sport-specific training usually begins. The early rehabilitation protocols were analyzed with regard to the following rehabilitation criteria (see Table 1): postoperative weight-bearing recommendations, restriction of range of motion (ROM), use of braces, recommended continuous passive/active motion (CPM/CAM), as well as the recommended start of rehabilitation training and specific training. Rehabilitation training was defined as any type of basic sports activity to restore coordination and muscle strength, such as ergometer, cycling, aqua jogging, general strength training, or crawling. Specific training included roadwork training, coordination, and proprioception training, as well as sports-specific training. Criteria for the rehabilitation progress such as the duration and time points of limitations and recommendations were documented. All categories, excluding rehabilitation or specific training, were analyzed up to three days postoperatively, seven days postoperatively, and then weekly until full weight bearing and full range of motion were allowed or until knee braces or further CPM/CAM training were no longer required.

Data analysis—Statistical analysis was performed with SPSS^®^ (Version 25, IBM, Armonk, NY, USA). Data are presented as mean ±SD or absolute and relative frequencies. Continuous data between two or more groups were compared with analysis of variance (ANOVA). Categorical data were compared using the chi-squared-test of independence. In case of significance, further post-hoc tests (according to Fisher’s least significant difference) were calculated. A probability (*p*) value of ≤0.05 was considered to be significant for each test. Graphical illustrations were generated with GraphPad Prism^®^ (Version 5.01, GraphPad Software, La Jolla, CA, USA) and Microsoft PowerPoint 2013^®^ (Microsoft Corporation, Redmond, WA, USA).

## 3. Results

### 3.1. Algorithm of Evaluation

In total, 620 protocols from 120 different orthopedic institutions were collected. A total of 33 (5.3%) of the 620 protocols described the early rehabilitation phase after isolated PCL-R. Twenty (60.6%) of the institutions, one of them a university medical center, provided stationary health care, thirteen (39.4%) were ambulatory. None of the PCL-R rehabilitation protocols were identical.

### 3.2. Progression of Weight Bearing and Range of Motion

**Figure 1:**
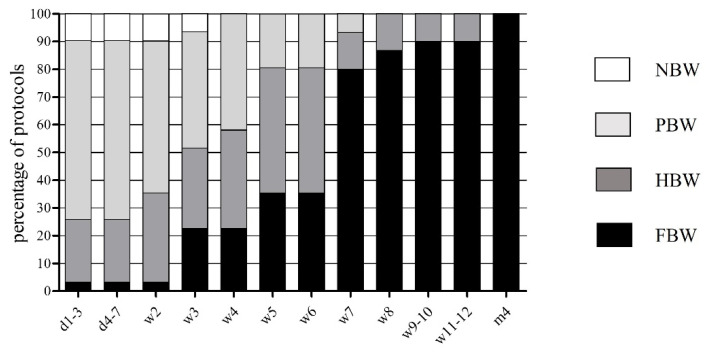
Early rehabilitation phase after PCL-R concerning weight bearing, expressed in percentage of protocols (%). NBW: no body weight, PBW: partial body weight, HBW: half body weight, FBW: full body weight, d: day, w: week.

**Figure 2:**
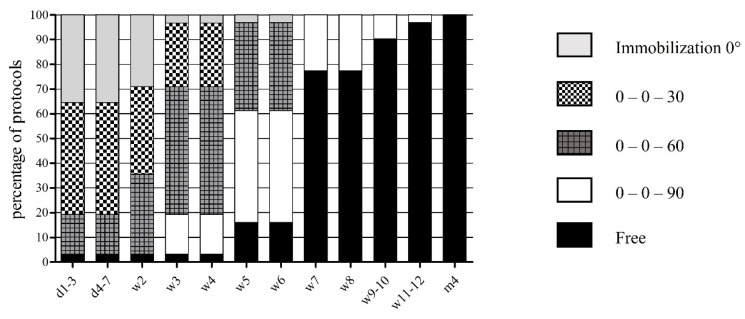
Early rehabilitation phase after PCL-R concerning range of motion (ROM), expressed in percentage of protocols (%). d: day, w: week.

Of the 33 PCL-R protocols, 31 contained detailed information on how to proceed with weight bearing and range of motion (ROM) (see Figure 1 and Figure 2). For the first postoperative week, recommendations ranged from ‘no body weight’ (n = 3; 9.7%) to ‘full body weight’ (n = 1; 3.2%), although most recommendations were ‘partial weight bearing’ (n = 20; 64.5%) or even ‘half body weight’ (n = 7; 22.6%). ‘Full body weight’ was recommended immediately after surgery in 1 (3.2%) protocol, after two weeks in 6 (19.4%) protocols, after four weeks in 4 (12.9%) protocols, after six weeks in 13 (41.9%) protocols, and, at the latest, after 12 weeks in 1 (3.2%) protocol.

In the first postoperative week, 11 (35.5%) of 31 protocols recommended 0° to 10° of flexion of the knee joint, 14 (45.2%) protocols up to 30°, and 5 (16.1%) protocols up to 60°. One (3.2%) protocol even allowed pain-adapted, but in principle unrestricted, flexion and extension from the beginning. The majority of orthopedic surgeons recommended a steady increase in ROM in steps of further 30° over the following weeks. After six weeks, the majority of protocols (n = 24; 77.4%) recommended release of the full range of motion in flexion, whereas 7 (22.6%) protocols set the limit at 90°. This limit was finally to be lifted after 9 weeks in 12.9%, after 13 weeks in 6.5%, and after 17 weeks in 3.2% of the protocols.

### 3.3. Use of Knee Braces

According to the post-treatment protocols, the restriction of ROM in case of full restriction of flexion was mostly ensured by the use of an adjustable knee brace or a posterior tibial support brace (PTS). Thirty (90.9%) of the thirty-three included protocols provided specific information on the type of brace and their wearing period. Each protocol that included information about braces (100%) recommended the use of a brace immediately after surgery, approximately one third (n = 9; 30.0%) a PTS, and two thirds (n = 21; 70.0%) an adjustable knee brace. The mean wearing period of a knee brace after PCL-R is 12.2 ± 2.2 weeks (range: 6 to 24 weeks).

### 3.4. Rehabilitation and Specific Training

A total of 27 (87.1%) of the 33 protocols contained specific recommendations on rehabilitation and specific training after PCL. Rehabilitation training was recommended at the earliest after 8 weeks in four (14.8%) protocols and at the latest after 4 months in nine (14.8%). The majority of protocols (n = 14; 51.9%) recommended starting rehabilitation training between the 8th and 12th postoperative week. Two (7.4%) out of twenty-seven protocols recommended starting specific training at the earliest after 12 weeks and 10 (37.0%) after 6 months and later.

## 4. Discussion

The early rehabilitation protocols after PCL-R of various institutes did not correspond with regard to individual rehabilitation procedures. Some protocols contained comparable procedures, yet no standardized and more individual and personalized procedures. The main consensus found in over 90% of the protocols was on wearing a knee brace during early rehabilitation. The recommended duration of wearing a brace for an average of 12.2 weeks was almost twice as long as after isolated anterior cruciate ligament reconstruction (6.8 weeks; *p* < 0.001) [23]. This finding shows how highly orthopedic surgeons value additional posterior tibial support and guidance of the knee joint during the first weeks and months after knee surgery. If the type of brace was indicated on the rehabilitation protocol, it was to a large extent a posterior tibial support brace. The main function of this brace is to take over the primary function of the PCL, which is to limit posterior tibial translation relative to the femur. The posterior tibial support should minimize traction forces on the freshly inserted graft. Another point that can be considered consensus is the fact that well over 90% of surgeons limit weight bearing to a maximum of half body weight, as well as clearly limiting the range of motion in flexion to a maximum allowed 60° for at least the first two weeks after surgery (see Figure 1 and Figure 2). These measures are intended to address the low biomechanical loading capacity of the inserted graft. The results clearly show that restriction of weight bearing and ROM after PCL-R is standard procedure in clinical practice.

However, the main finding of our study was the wide range of different rehabilitation protocols after PCL-R, in particular regarding the progression of weight bearing and ROM of the knee joint. Previous researchers did also not find any relevant consensus when systematically reviewing post-treatment recommendations after PCL-R [10,25,26]. The lack of both standardization and scientific evidence is principally a lack for each medical treatment procedure but opens the way for individualized rehabilitation protocols. Surgeons’ experience and the activity level of the existing patient collective after PCL-R provide the basis for a personalized but not a standardized rehabilitation protocol; however, surgical techniques still differ significantly and thus play an important role in early post-surgical rehabilitation [4,20,21]. In addition to the preoperative activity level and the surgical technique used, the individual goal of rehabilitation must also be taken into account when planning the rehabilitation phase. This should already be formulated together with the patient preoperatively and also be included in the indication for surgical treatment. These individual goals can range from ‘regaining the stability of the knee joint and mobility necessary for everyday life’ to ‘return to high-performance sports’. Accordingly, the training plans must be adapted to this, and a longer rehabilitation phase must be scheduled the higher the performance goal.

The fact that different post-treatment protocols—based on fixed postoperative time points—partly differ significantly in their respective rehabilitative criteria is not PCL-specific but also applies to other surgical therapies such as repair of the meniscus [27,28], the ACL [23,29], the medial patellofemoral ligament [30,31], or cartilage [24].

Of course, the range of different recommendations seen from the results cannot be attributed solely to the presumption that an individualized approach is preferred. In this respect, the presented results cannot be generalized. One of the limitations that must be blamed for this is the fact that the investigated protocols do not take into account the time of indication for surgical therapy (surgical therapy as primary therapy versus secondary after unsuccessful conservative therapy). Furthermore, due to the retrospective design of the descriptive analyses of rehabilitation protocols of different medical settings, individual clinical outcome or complications during the rehabilitation phase were not included in the assessment of rehabilitation protocols in the context of this study. The same applies to the lack of sociodemographic data such as sex, age, or the pre-operative activity level of the patients. Furthermore, there is no information about the surgical technique applied. However, this study shows the current clinical routine and daily practices of orthopedic surgeons and therefore the current status of postoperative care after PCL-R.

Standards in rehabilitation should obviously not be presented in the form of timeline-based protocols and schedules that refer to the time since surgery; here, a much more differentiated approach seems to be required. The first step towards a patient-centered, personalized rehabilitation standard would be to disregard the notion that ‘time since surgery’ is a fixed criterion for reaching the next step, such as increasing weight bearing or the release of ROM. A scheduled rehabilitation protocol may suit one patient but may proceed too fast for another with a possible negative clinical outcome. Vice versa, however, professional athletes may achieve their rehabilitation goals more quickly than recreational athletes with a lower preoperative activity level. By setting rehabilitation goals rather than time points, a patient’s deficits and potentials can be adequately addressed. This way, post-surgical rehabilitation becomes more personalized and tailored to the individual patient. A similar approach has been proposed for the far more commonly performed reconstruction of the ACL [32] and meniscal repair [33]. For PCL-R, detailed rehabilitation standards already exist that do not contain any time points, but different ‘phases’ separated by ‘goals’, which have to be achieved before entering the next rehabilitation ‘phase’ [25]. This concept also means that rehabilitation does not end after a certain period of time, for instance, after three or six months, but best after the knee joint has regained and maintained its full functionality and the patient has returned to sports activity.

## 5. Conclusions

The only consensus in the currently existing rehabilitation protocols after PCL-R was on wearing a brace after knee surgery as well as the restriction of weight bearing and ROM immediately post-surgery. However, the wide range of different rehabilitation procedures, especially with regard to the progression of weight bearing and ROM, shows that a standardized post-surgical rehabilitation protocol is not available in clinical practice. Rather, individual rehabilitation seems to be the current solution in daily routine, depending on the time of indication, the surgical technique applied, and most importantly individual patient factors such as pre-operative activity level and the desired goal at the end of the rehabilitation phase.

## Figures and Tables

**Table 1:** Criteria of early rehabilitation.

Criteria of Early Rehabilitation	
Weight bearing	No body weight (NBW)
	Partial body weight (PBW; loading up to 20 kg)
	Half body weight (HBW; loading more than 20 kg)
	Full body weight (FBW; unlimited loading)
Range of motion (extension/flexion)	Immobilization 0°
	0–0–30°
	0–0–60°
	0–0–90°
	Free
Use of braces	Yes/No
	Recommended wearing time (weeks)
Continuous passive/active motion	No CPM/CAM recommended
	CPM recommended
	CAM recommended
Start of rehabilitation training	Weeks after surgery
Start of specific training	Weeks after surgery

## Data Availability

The data presented in this study are available on request from the corresponding author. The data are not publicly available due to privacy reasons.
